# Medical students’ preparedness for professional activities in early clerkships

**DOI:** 10.1186/s12909-017-0971-7

**Published:** 2017-08-22

**Authors:** Josefin Bosch, Asja Maaz, Tanja Hitzblech, Ylva Holzhausen, Harm Peters

**Affiliations:** Dieter Scheffner Center for Medical Education and Educational Research, Department of Study Affairs, Charité – Universitätsmedizin Berlin, Berlin, Germany

## Abstract

**Background:**

Sufficient preparedness is important for transitions to workplace participation and learning in clinical settings. This study aims to analyse medical students’ preparedness for early clerkships using a three-dimensional, socio-cognitive, theory-based model of preparedness anchored in specific professional activities and their supervision level.

**Methods:**

Medical students from a competency-based undergraduate curriculum were surveyed about preparedness for 21 professional activities and level of perceived supervision during their early clerkships via an online questionnaire. Preparedness was operationalized by the three dimensions of confidence to carry out clerkship activities, being prepared through university teaching and coping with failure by seeking support. Factors influencing preparedness and perceived stress as outcomes were analysed through step-wise regression.

**Results:**

Professional activities carried out by the students (*n* = 147; 19.0%) and their supervision levels varied. While most students reported high confidence to perform the tasks, the activity-specific analysis revealed important gaps in preparation through university teaching. Students regularly searched for support in case of difficulty. One quarter of the variance of each preparedness dimension was explained by self-efficacy, supervision quality, amount of prior clerkship experience and nature of professional activities. Preparedness contributed to predicting perceived stress.

**Conclusions:**

The applied three-dimensional concept of preparedness and the task-specific approach provided a detailed and meaningful view on medical students’ workplace participation and experiences in early clerkships.

## Background

Early clerkships represent the first major transition in medical training, i.e. from university-based classroom to clinical workplace learning [1, 2]. They offer unique learning opportunities for students through active participation in workplace activities characteristic of the medical profession [1, 3–6]. Furthermore, clerkship placements play an essential role for medical students’ identity formation and professional socialisation [1, 7]. In turn, inadequate preparation and preparedness for clerkships is associated with stress and anxiety for medical students starting out [8–10], which may both impede the transition and hamper learning though participation within the clinical setting. It may also interfere with patient safety or increase hospital costs [10–12].

The information on medical students’ preparedness for early clerkships is limited. A few educational research studies only have explored this phase [13–23]. In those studies, preparedness has been inconsistently defined and conceptualized and has been studied mostly in a global way, for instance for the clerkship as a whole, or with broad themes like contact with patients, medical knowledge or general skills [13, 15, 17, 19]. A differentiated conceptualisation of preparedness as well as a task-specific analysis offer the potential to advance our understanding of medical students’ preparedness for early clerkships and to better inform curricular developers about preparation needed for easing the transition and improving students’ workplace participation and learning.

**Fig. 1:**
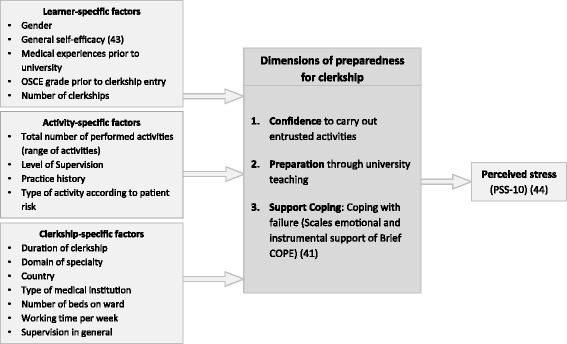
Preparedness concept and study design with variables included

Preparedness and its conceptualisation have recently gained increasing attention in medical education [24]. For the purpose of this study, we developed a three-dimensional theoretical concept of preparedness which was derived from two lines of research. Organizational psychology bases preparedness on socio-cognitive theory [25] and defines it as “a goal state of readiness to respond to uncertain outcomes” [26]. It consists of two dimensions [27, 28]. One dimension is the confidence in one’s own ability to implement or carry out required actions, and can be understood as domain-specific self-efficacy. The other dimension is the capability to deal with failure and setbacks. We focus on coping with failure by means of searching for support as active support seeking has been shown to be related positively to both task mastery and dealing with transitions and their challenges [9, 29]. As third dimension, we included students’ self-evaluation of being prepared by university teaching (see Fig. 1). This builds on medical education research in which preparedness is generally defined as the students’ evaluation of how prepared they feel by medical school (e.g. [11, 30]) and whether or not they learned “the right things” [24].

Medical students’ preparedness is likely influenced by a variety of factors which have been investigated foremost among medical graduates. They can be categorized into personal characteristics [9–11, 15, 30], aspects of the working environment [11], as well as type and duration of education [9–11, 15, 30–33].

Analysis of students’ learning during early clerkships has been found to be challenging. It seems to occur within a so-called “black box” and existing findings show a high variation in workplace involvement and supervision [3, 4, 34, 35]. A novel perspective to capture workplace participation in clerkships is offered by analysing which professional activities students actually carry out and how they are supervised. Both constructs – professional activities and supervision levels – have recently been introduced and operationalized as key elements of the framework of Entrustable Professional Activities (EPAs, [36, 37]). Professional activities represent authentic units of work of a profession that learners may carry out in the clinical workplace. Supervision levels indicate the level of autonomy which the learner is assigned to by a supervisor for a certain professional activity. This simple matrix aligns in a meaningful manner with the students’ learning trajectory in the workplace, i.e. increasing the range of activities and decreasing the supervision needed.

This approach can also improve our understanding of clerkship preparedness. The type of activity and expected supervision level represent key determinants of a student’s perceived preparedness for the task. For instance, preparedness will likely be different if a challenging task is supposed to be carried out in co-activity with a supervising physician, or without the physician being in the room. Consequently, a task- and supervision-specific representation of students’ preparedness can provide tangible information for curriculum developers and program adjustment. Previous research has explored clerkship students’ preparedness for a few specific medical tasks, for instance history taking and physical examination [13, 15, 17, 19, 38]. However, task preparedness was not analysed in relation to a specified level of supervision and rarely surveyed on a comprehensive range of professional activities.

The purpose of this study is to improve our understanding of medical students’ preparedness for workplace participation in early clerkships and to explore how a task-specific approach can yield insights for curriculum improvement to ease the transition. The following research questions will be addressed:

1. Which professional activities do medical students carry out during early clerkships and what are the perceived corresponding supervision levels?
2. How well prepared do students feel for professional activities when they perform tasks during early clerkships?
3. What factors influence students’ preparedness in early clerkships?
4. How does students’ preparedness influence their perceived stress in the course of the early clerkship?

## Methods

### Participants and setting

Medical students from the competency-based undergraduate medical curriculum (UME) at the Charité – Universitätsmedizin Berlin (Charité), Germany were investigated in a cross-sectional study. The curriculum was developed and structured on the basis of a pre-defined outcome catalogue comparable to the CanMeds framework [39]. It represents a 6 year program and is taught in 40, vertically and horizontally integrated modules. Real patient-based learning takes place right from the beginning of the studies, every other week in year 1 and weekly in year 2. This is paralleled by biweekly communication courses and peer-assisted skills training in a skills lab. After year 2, students have to pass an objective structured clinical examination before they can enter year 3. Starting in year 3, students have to accomplish 4 months of obligatory clerkships during the semester breaks. The clerkships are generally of 4 weeks in length and the students themselves organize the placements, i.e. the hospital and type of clinical ward. The goal of the clerkship is to allow for workplace participation. No specific learning objectives are set for the clerkships.

In the first 4 weeks of the winter term 2013/2014, an online questionnaire was sent to all medical students (*N* = 755) starting their 3rd-and 4th-year, via the evaluation software Evasys (Electric Paper Evaluationssysteme GmbH, Lüneburg, Germany). It requested information about the clerkships performed during the preceding semester break, which were their first clerkships at this point in the program (early clerkships).

### Measures

**Fig. 2:**
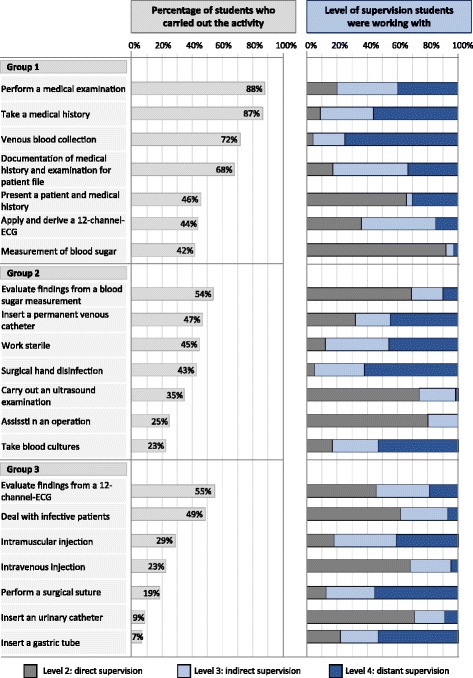
**a** Percentage of students who carried out the activities per task during the course of their clerkship. **b** Percentage of students who were working under the indicated supervision level per activity (*N* = 147 in a + b)

Central to the questionnaire was a list of 21 professional clerkship activities. The list was derived from the German catalogue of learning objectives for practical skills for UME [40]. The activities were purposely selected by members of the Charité curriculum development team and through pilot testing with clerkship students, to represent activities typically performed by early clerkship students (Fig. 2). In a survey with 20 physicians, the 21 activities were classified into three activity types according to the possible risk for a patient if the task were performed by a new, yet not sufficiently known medical student without direct supervision (see Fig. 2, group 1 corresponds to low patient risk, group 3 to higher patient risk).

In the online questionnaire, the students reported whether they had performed each of the tasks or not during their previous clerkship. Each professional activity was assessed for *practice history* (ratio of activities performed for the first time during the clerkship versus performed already before) and *level of perceived supervision* by a physician (according to the level of proficiency for EPAs, [37]). Supervision levels were: (1.) acting with direct, proactive supervision, (2.) acting with indirect, re-active supervision, and (3.) acting with supervision not readily available, but with distant supervision and oversight. *Range of tasks* was calculated as the total number of activities indicated out of the 21 professional activities.

We measured preparedness on three dimensions. The first dimension was operationalized as “confidence to carry out clerkship activities” (*confidence*) and the second as “being prepared through university teaching” (*preparation through university*). We assessed both in an activity-specific manner, i.e. through one item for each of the professional activities﻿; referring to the first performances during the clerkship (e.g. “I felt confident to perform a physical examination” and “I felt qualified by the courses at university to take a medical history.”) on a 6-point Likert scale (1 = strongly disagree, 6 = strongly agree). A score for confidence and preparation through university was calculated as the sum of each activity-specific evaluation divided by the number of activities performed by a student. We operationalized the third dimension “coping with failure” through the scale *support coping* of the Brief COPE [41] in the German version by Knoll et al. [42]. It comprises the subscales instrumental and emotional support and is calculated as the mean of the four items addressing support searching behaviour in case of difficulty. They were measured on a 4-point Likert-scale (1 = not at all, 4 = very much).

Based on a literature review, factors associated with preparedness were identified (see introduction) and grouped into learner-specific, activity-specific and clerkship-specific variables (see Fig. 1). Learner-specific and clerkship-specific factors were surveyed mostly via single items. General self-efficacy was measured using the 10-item scale by Schwarzer and Jerusalem [43]. Clerkship-specific variables were included to control for different clerkship settings.

Perceived stress as an outcome was surveyed using the PSS-10 scale [44].

Depending on how many professional activities were indicated, the questionnaire consisted of a minimum of 70 items and a maximum of 170. The questionnaire was pilot-tested for comprehension and performance by clerkship students.

### Statistical analysis

Research questions 1 and 2 were tested via descriptive statistics and analysis of variance to compare activity groups and supervision levels. Research questions 3 and 4 were examined via correlation and step-wise regression. They allow us to quantify the relative contribution of single independent variables or groups of variables to the total variance of the model. Each preparedness dimension was first correlated with the influencing factors. In the regression analysis, each preparedness dimension represented the dependent variable once. Variables correlating significantly with the preparedness dimensions were mean-centred and entered group-wise as independent variables. *Perceived stress* was set as a dependent variable in an additional regression analysis. In a first step, the correlating preparedness dimensions were included to test the unique contribution of preparedness on the outcome. Influencing variables correlating with *perceived stress* were entered during the subsequent steps.

Statistical analysis was executed using SPSS 23.0 (IBM SPSS Statistics, Armonk, NY, USA). A *p*-value of α <0.05 was considered to be significant.

## Results

### Participants

Out of 775 students invited to participate, data from 150 students (19.4%) were obtained. Of those, three participants were excluded due to insufficient data (*n* = 147; 19.0%). Since only about two thirds of the invited students had actually carried out a clerkship during the preceding semester break, the adjusted response rate is 28.3%. The gender proportion of 70.1% women corresponds to that of the general student population here. The mean age of the participants was 23.6 years (*SD* = 3.5).

Cronbach’s alpha for all scales used ranged between α = 0.83 and α = 0.90, indicating a good internal consistency.

### Professional activities performed by medical students

Figure 2a depicts a task-specific overview on the frequency of students who carried out the 21 selected professional tasks during their clerkships. On the average, medical students performed about 9 different professional activities (*M* = 9.2; *SD* = 3.7). Group 1 activities covered the largest part of activities (52%), followed by group 2 (28%) and group 3 (21%) activities (*F*[145,2] = 84.6; *p* < 0.0001). Out of group 1 activities, 15% were performed for the first time; out of group 2 and 3 activities, 30% were new to the students. Beyond the activity list provided, students reported that they had participated in other activities, for instance punctures, wound treatment and intubation.

The degree of supervision varied between activities, which is depicted in Fig. 2b for each task. Overall, about one third of all tasks were conducted either under direct supervision (32%), indirect supervision (29%), or distant supervision (33%); 6% were supervised in a different way, or supervision was not indicated. Analysis by ANOVA revealed an interaction between supervision and group of activity (*F*[117,4] = 28.2; *p* < 0.0001) indicating that all three activity groups were supervised differently. Group 1 activities were most often supervised distantly (43% of group 1 activities) and least often directly (20%). Group 2 and 3 activities were most often supervised directly (43% of group 2 activities; 46% of group 3 activities). Group 3 activities were least often supervised distantly (18%), whereas group 2 activities were equally supervised indirectly (25%) and distantly (28%).

### Degree of preparedness

**Fig. 3:**
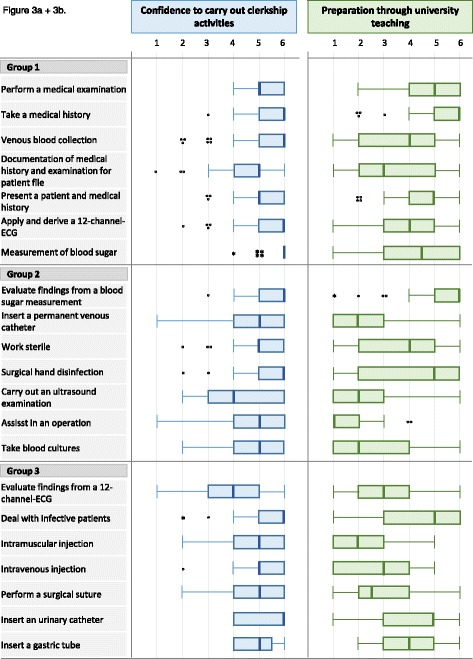
**a** Confidence to carry out the clerkship activities; per activity on 6-point Likert-scale. **b** Preparation through university teaching; per activity on 6-point Likert-scale. Legend: 1 = strongly disagree, 6 = strongly agree and *N* = 147 in a + b

A task-specific representation of students’ *confidence* and *preparation through university* to carry out the task is depicted in Fig. 3. Overall, the students reported a relatively high *confidence* for the tasks (*M* = 5.0; *SD* = 0.6), with lower ratings for performing an ultrasound or evaluating ECG findings. *Preparation through university* (*M* = 3.9; *SD* = 1.0) showed a more heterogeneous picture within and between tasks, with negative ratings (median 2 or lower) for one fifth of the 21 activities. *Confidence* and* preparation through university* aligned well for some tasks (history taking, ultrasound examination), while others were deviating (assistance in an operation, insertion of a permanent venous catheter).

*Confidence* differed between all three activity groups (*F*[119,2] = 20.7; *p* < 0.0001) with students being most confident for group 1 activities (*M* = 5.2; *SD* = 0.6) and least confident for group 3 activities (*M* = 4.6; *SD* = 1.1). The same pattern was found for *preparation through university* (*F*[119,2] = 76.7; *p* < 0.0001; group 1: *M* = 4.4; *SD* = 1.0; group 3: *M* = 3.2; *SD* = 1.3). Preparedness in the different supervision levels was explored via pairwise t-tests, as only half of the students were supervised on all three levels. Both *confidence* and *preparation through university* were highest for distantly supervised activities (*confidence*: *M* = 5.4; *SD* = 0.6; *preparation through university*: *M* = 4.3; *SD* = 1.2) compared to directly (*confidence*: *M* = 4.7; *SD* = 1.0; *preparation through university*: M = 3.4; SD = 1.3) or indirectly supervised activities (*confidence*: *M* = 4.9; *SD* = 0.9; *preparation through university*: *M* = 3.6; *SD* = 1.4; *p* < 0.0001).

The preparedness dimension to deal with failure and setbacks was analysed globally. The clerkship students regularly sought support (*M* = 2.4; *SD* = 0.8) in case of difficulty. Female students (*M* = 2.5; *SD* = 0.9) showed a higher rate of *support coping* than male students did (*M* = 2.2; *SD* = 0.8; *t*[142] = −2.3; *p* < 0.05).

### Factors influencing preparedness

In the regression analysis, the influencing factors accounted for about one fifth to one quarter of the variance of the preparedness dimensions (17–26%, see Table 1 for the coefficients of the respective last step).

**Table 1:** Stepwise regressions of preparedness dimensions on influencing factors﻿﻿﻿﻿﻿

1. 1. Confidence to carry out clerkship activities					
Model	Step	R^2^	ΔR^2^	F	
	1 learner	0.14	0.14	22.72***	
	2 learner + activity	0.25	0.11	15.53***	
	3 learner + activity + clerkship	0.25	0.00	11.88***	
Regression coefficients (step 3)	Variables	B	SE	β	t
Learner	Self-efficacy	0.48	0.12	0.29	3.93***
Activity	Ratio first time	−0.01	0.00	−0.30	−3.93***
	Range of activities	0.04	0.01	0.24	3.04**
Clerkship	General supervision	0.05	0.05	0.07	0.98
2. 2. Preparation through university teaching					
Model	Step	R^2^	ΔR^2^	F	
	1 learner	0.10	0.10	6.21**	
	2 learner + activity	0.17	0.07	5.45***	
	3 learner + activity + clerkship	0.26	0.09	5.27***	
Regression coefficients (step 3)	Variables	B	SE	β	t
Learner	Self-efficacy	0.46	0.20	0.20	2.30*
	Medical experiences prior to university	−0.20	0.19	−0.09	−1.01
	Number of clerkships	−0.25	0.11	−0.20	−2.31*
Activity	Ratio group 1 activities	0.01	0.01	0.21	1.81
	Ratio group 2 activities	0.00	0.01	0.04	0.29
Clerkship	More than 25 beds on the ward	−0.29	0.20	−0.13	−1.44
	General supervision	0.17	0.08	0.19	2.13*
3. 3. Support Coping					
Model	Step	R^2^	ΔR^2^	F	
	1 learner	0.04	0.04	5.36*	
	2 learner + activity	0.17	0.13	9.80***	
Regression coefficients (step 2)	Variables	B	SE	β	t
Learner	gender	0.28	0.14	0.16	2.01*
Activity	Ratio first time	0.01	0.00	0.31	3.91***
	Range of activities	0.03	0.02	0.13	1.69

Legend: *Individual* individual fact﻿ors, *clerkship* clerkship-specific factors, *activity* activity-specific factors, *B* unstandardised regression coefficient, *SE* Standard error, *β* standardi﻿sed regression coefficient

***p < 0.0001, **p < 0.01, *p < 0.05

*Confidence* was predicted by self-efficacy, ratio of activities performed for the first time and the range of activities (*R* ^*2*^ = 0.25; *F*[4146] = 11.88; *p* < 0.0001). Students felt more confident when they had more self-efficacy, performed a small amount of activities for the first time and when they performed many of the 21 activities.

The variance of *preparation through university* was accounted for by self-efficacy, the number of clerkships and the supervision in general (*R* ^*2*^ = 0.26; *F*[7114] = 5.27; *p* < 0.0001, see Table 1). Students felt better prepared through university when they had a higher self-efficacy, did more prior clerkships, and perceived their general supervision as better.

S*upport coping* was predicted by female gender. In addition, students who performed a higher percentage of activities for the first time more frequently looked for support. (*R* ^*2*^ = 0.17; *F*[3144] = 9.80; *p* < 0.001, see Table 1).

### Outcome of preparedness

Students did not indicate a high level of stress (*M* = 11.2; *SD* = 6.1, whereby a maximum of 40.0 represents high stress). Significant predictors of perceived stress were support coping, self-efficacy and general supervision (*R* ^*2*^ = 0.48; *F*[7144] = 18.39; *p* < 0.0001; see Table 2). Students who showed higher support coping, had a lower self-efficacy. F﻿urthermore, students ﻿who perceived their general supervision as poor, felt more stressed during their clerkship.

**Table 2:** Hierarchical regressions of perceived stress on influencing factors and preparedness dimensions﻿

Perceived stress					
Model	Step	R^2^	ΔR^2^	F	
	1 preparedness	0.16	0.16	8.82***	
	2 preparedness + learner	0.27	0.11	12.84***	
	3 preparedness + learner clerkship	0.47	0.20	24.76***	
	4 preparedness + learner + clerkship + activity	0.48	0.01	18.39***	
Regression coefficients (step 4)	Variables	B	SE	β	t
Preparedness	Confidence	−0.11	0.07	−0.12	−1.60
	Support Coping	0.04	0.01	0.20	2.99**
	Preparation through university	−0.06	0.05	0.09	1.19
Learner	Self-efficacy	−0.50	0.10	−0.33	−4.98***
Clerkship specific	General supervision	−0.27	0.04	−0.45	−6.88***
Activity specific	Ratio first time	0.00	0.00	0.07	1.08
	Ratio group 2 activities	0.00	0.00	0.10	1.46

Legend: *Individual* individual fact﻿ors, *clerkship* clerkship-specific factors, *activity* activity-specific factors, *B* unstandardised regression coefficient, *SE* Standard error, *β* standardi﻿sed regression coefficient

***p < 0.0001, **p < 0.01, *p < 0.05

## Discussion

This study introduces and explores a three-dimensional, task-specific concept of preparedness. Its results provide detailed and illuminating insights on how early clerkship students participate in clinical settings and how they experience their transition to clinical workplace learning. Students in our sample participated in a wide range of professional activities and worked under a broad range of supervision levels. They felt variably confident to perform the activities and all on a high level. In turn, the evaluations of preparation through university teaching were highly diverse between students and activities and may reveal gaps in the preceding curriculum. In case of difficulty to perform a task, students of this study sample regularly searched instrumental and emotional support. For the understanding of workplace participation and perceived preparedness in this study, it is important to bear in mind that no task-specific learning objectives are set for these early clerkships. Under these circumstances, students’ workplace participation and their individual learning pathway offers the opportunity to develop naturally according to the tasks to be done in a specific clinical setting, the students’ perceived ability for the task and the arrangement and coordination with the supervisor on the appropriate supervision level.

### Professional activities and supervision

Overall and in line with other reports, students in this study themselves differed greatly in regard to the number and type of clerkship activities [3, 4, 34, 35], which is likely a reflection of the differences in clerkship settings. Taking a patient’s history and performing a physical examination constituted the main tasks in our early clerkships. Besides those basic activities, students in this study sample took part also in more demanding tasks, e.g. evaluating findings from an ECG or giving injections. Interestingly, about one third of the activities were performed with the supervisor observing the student. During the performance of another third of activities, the supervisor was easily available. These may be seen as positive findings in terms of students’ learning opportunities, feedback and assessment [4, 35, 45], and are in divergence with other reports where direct supervision is more often lacking than available [3, 4, 35, 46]. In addition, the students reported executing about one third of their activities under distant supervision. This applied to a great extent to clerkship activities associated with lower patient risk (group 1), which comprised about half of the students’ activities. About one quarter of the activities were of medium or higher risk (groups 2 and 3), and were most often directly supervised. In our study sample, this seems to reflect appropriate supervision behaviour and aligns reasonably with the students’ learning trajectory and progress. However, some students reported carrying out higher risk activities without supervision readily available (e.g. intramuscular injection). This may have increased patient risk and should be taken into consideration by supervisors.

Chen et al. [1] recently proposed that undergraduate students should be supervised up to the indirect supervision level, which is in line with the present study. Although clinical physicians and supervisors essentially make entrustment decisions in clinical situations all the time, this process is not yet formalized and explicit and therefore is difficult to survey. In our clerkship field situation, we used the students’ self-evaluation on how supervision was enacted in practice during the first clerkship activities and not evaluations directly provided by the supervising physicians.

### Preparedness

Our results concerning a three dimensional conceptualisation of preparedness noticeably substantiate and expand former studies [13, 15, 17, 38] and draw a task-specific picture of the concept for activities beyond history taking and performing a physical examination. The students in our sample felt most confident and most prepared by university teaching for professional activities with low patient risk (e.g. medical examination) and least for activities associated with higher patient risk (e.g. evaluate findings from an ECG). Nevertheless, preparedness was not homogenous within these groups, but in part quite diverse. Considering the early stage during their undergraduate education, it seems reasonable that students first build up their preparedness for basic medical activities before progressing to more demanding tasks later on.

The responding students also differentiated their preparedness as a function of the supervision level itself. Both confidence and preparation through university teaching increased as perceived supervision decreased. Together with the above-mentioned finding that they most frequently performed low risk activities under distant supervision and high risk activities under direct supervision, this draws a coherent picture of students’ clerkship activities and preparedness. It remains an open question whether the students were formally allowed to practice under distant supervision because they were actually evaluated as proficient, or if they felt more confident because they were entrusted on a higher level out of situational needs.

For the students sample investigated it has to be taken into account that confidence generally seemed to be very high and that preparation through university teaching was always ranked lower. This aligns with findings that people generally tend to estimate their own capabilities above average. On the one hand, this is useful, as a high self-efficacy strengthens persistence and advancement in learning [47]. On the other hand, it can be dangerous to have a student who is overconfident and works beyond the scope of his or her limits, particularly in medicine. Nevertheless, students seemed to align their confidence in relation to the nature of activity and supervision, which indicates that they are, at least on a basic level, able to reasonably self-evaluate their own abilities.

### Factors influencing preparedness

Employing this concept of preparedness yielded interesting insights on how it is influenced by learner-, activity- and clerkship-specific factors. Our study revealed that about one fifth to one quarter of the inter-individual differences of each dimension can be explained. Learner-specific characteristics and the nature of the activity had an effect on all three dimensions, whereas clerkship features only influenced preparation through university teaching.

Learner-specific characteristics influencing preparedness included general self-efficacy, gender, as well as the amount of prior clerkship experience. Students with a high general self-efficacy perceived themselves as better prepared, as shown previously [27, 31]. They were more confident and perceived their university teaching as a more helpful preparation for their clerkships. So far, preparedness has been found to be independent of gender [11, 30]. Here, it was linked to support coping, which is stronger for female students. Having completed more previous clerkships reduced preparedness through university teaching. Ochsmann et al. [11] did not find this relation in their data, possibly due to the fact that they studied university graduates and not students. Possible explanations are that the students may have internalized the knowledge gained during prior early clerkships and might not be able to consciously distinguish where they learned something, or that they start to recognize deficiencies in their university education in light of their own workplace experiences.

Activity-specific factors were found to be important for the degree of preparedness. If students performed a high percentage of activities for the first time, they felt less confident and sought support to cope with difficulties more often. Exposure to professional activities during studies or prior clerkships thus enhanced preparedness according to expectations [9]. A larger range of activities increased students’ confidence. Of course, our list of 21 activities is not exhaustive and can only be an approximation of the actual range of tasks students performed. In any case, one’s own mastery experiences in the workplace are the most important driver of students’ self-efficacy [25, 48], and because confidence constitutes a domain-specific self-efficacy, this is in line with expectations.

Among clerkship-related factors, only the perceived general supervision appeared to influence preparedness in this study. It had an effect on preparation through university in a way that students who indicated a high general supervision in their clerkships were also better prepared by university teaching. This goes along with a study by Cave et al. [30]; nonetheless, it might be somewhat counterintuitive that supervision quality itself does not affect confidence but university preparation.

### Outcome of preparedness

Overall, the participating clerkship students did not feel too stressed. Their stress level corresponded to the average stress level of the normal population [49]. Half of this variance could be predicted by our model. Students with lower self-efficacy [50] and lower general supervision quality experienced more stress, which represents a starting point for prevention. Students who more often looked for help in case of difficulties felt more stressed during their clerkships. One possible explanation might be that if students generally do not feel supervised enough in their clerkship, they feel too stressed to ask for help in case of difficulty. Our results are in line with previous findings showing a relation between preparedness and stress level during the transition to early clerkships [30, 51].

### Limitations

For a generalization of the results here, it should be taken into consideration that our study investigated students from only one university. The investigated sample was not randomly drawn but based on self-selection of volunteering students who were willing to participate in the survey. This may cause bias in a way that students with certain characteristics (e.g. those caused by stress level, confidence, motivation, experiences during clerkships, work besides university, having children) did not take part in the first place. Another possible cause for sample bias is the low response rate. However, in email surveys the average response rate is about 30% [52], which corresponds to our adjusted response rate. Moreover it has to be mentioned critically that our study is one which addresses retrospective self-reported variables. We are aware of problems that may arise from subjective evaluations. Nevertheless, self-assessment is an adequate way to assess subjective cognitive constructs such as preparedness. It could be shown that a high preparedness is associated with good performance [12]. Furthermore, we asked the students subsequent to their clerkships how they felt at that time, which may cause hindsight bias. Additionally, we investigated medical students from a competency-based UME curriculum. Therefore our results might not be generalized to cover students from other curricula, coming for instance from a traditional discipline-based program. Derived from these limitation, future studies should expand the approach of this study to more institutions, different types of UME, full cohorts of clerkship students, inclusion of evaluation by supervising physicians and sampling of the preparedness-related information during the clerkships.

## Conclusions

Our study proposed a three-dimensional concept of preparedness offering a deeper understanding of the construct, its influences and outcomes. The task-specificity and insights in supervision levels of two preparedness dimensions allow us to draw a detailed and meaningful picture with regard to students’ learning trajectory in the clinical workplace. This may also help to identify curricular gaps and starting points for specific improvements.
